# Supplementary material for: Dominance of Influencing Factors on Cooling Effect of Urban Parks in Different Climatic Regions
Source: Int J Environ Res Public Health. 2022 Nov 22;19(23):15496. doi: 10.3390/ijerph192315496 (PMC9735790; doi:10.3390/ijerph192315496)
Supplement: Supplementary file 1 [file ijerph-19-15496-s001.zip › Supplementary Materials.pdf]

Table S1. Image information of Landsat used in the study.

Number	Images of Landsat	Images date	Path/Row	City	Central latitude and longitude (N, E)°
1	LC81270322020164LGN00	2020-06-12	127/32	Baotou	40.32010, 110.54385
2	LC81230322019229LGN00	2019-08-17	123/32	Beijin	40.31935, 116.72925
3	LC81180302019178LGN00	2019-06-27	118//30	Changchun	43.17069, 125.38215
4	LC81230402019229LGN00	2019-08-17	123/40	Changsha	28.86051, 113.46922
5	LC81290392019223LGN00	2019-08-11	129/39	Chengdou	30.29665, 104.57204
6	LC81280392019232LGN00	2019-08-20	128/39	Chongqing	30.29538, 106.11640
7	LC81190332020172LGN00	2020-06-20	119/33	Dalian	38.89153, 122.45107
8	LC81250322020166LGN00	2020-06-14	125/32	Datong	40.31979, 113.63201
9	LC81270322021214LGN00	2021-08-02	127/32	Ordos	40.32010, 110.54385
10	LC81190422020204LGN00	2020-07-22	119/42	Fuzhou	25.98381, 118.91911
11	LC81350322019217LGN00	2019-08-05	135/32	Jiayuguan Jiuquan	40.31945, 98.18717
12	LC81180282021247LGN00	2021-09-04	118/28	Harbin	46.01486, 126.40228
13	LE71190392020228EDC00	2020-08-15	119/39	Hangzhou	30.31701, 119.92112
14	LC81260322019218LGN00	2019-08-06	126/32	Hohhot	40.32008, 112.09242
15	LC81310352020208LGN00	2020-07-26	131/35	Lanzhou	36.03314, 103.06297
16	LC81210402019231LGN00	2019-08-19	121/40	Nanchang	28.85922, 116.55961
17	LC81200382018157LGN00	2018-06-06	120/38	Nanjin	31.73167, 118.86952

18	LC81180382020229LGN00	2020-08-16	118/38	Shanghai	31.73142, 121.95593
19	LC81180392020229LGN00	2020-08-16	118/39	Shanghai	30.29629, 121.57527
20	LC81190312020204LGN00	2020-07-22	119/31	Shenyang	41.74560, 123.36928
21	LE71220442019166EDC00	2019-06-15	122/44	Shenzhen Guangzhou	23.11923, 113.52740
22	LC81250342019227LGN00	2019-08-15	125/34	Taiyuan	37.46226, 112.75678
23	LC81220332020241LGN00	2020-08-28	122/33	Tianjin	38.89141, 117.82282
24	LC81230392020216LGN00	2020-08-03	123/39	Wuhan	30.29636, 113.84591
25	LC81420302020237LGN00	2020-08-24	142/30	Urumqi	43.17158, 88.31539
26	LC81270362019225LGN00	2019-08-13	127/36	Xi'an	34.60029, 108.83352
27	LC81290332019223LGN00	2019-08-11	129/33	Yingchuang	38.89200, 107.01115
28	LC81240362020239LGN00	2020-08-26	124/36	Zhengzhou	34.59898, 113.46792

Table S2. Data sources and explanation in this study.

Data	Resolution	Sources	Time series
Park boundary vector	1.0 m	Bigemap GIS Office	2019 and 2020
Landsat Images	30 m	USGS(https://earthexplorer.usgs.gov)	2019 and 2020
Precipitation	1000 m	National Earth System Science Data Center (http://www.geodata.cn)	2020
Land cover/ Land use	30 m	Earth Big Data Science Engineering Data Sharing Service System (https://data.casearth.cn)	2020
DEM	30 m	NASA(http://atmcorr.gsfc.nasa.gov)	2020

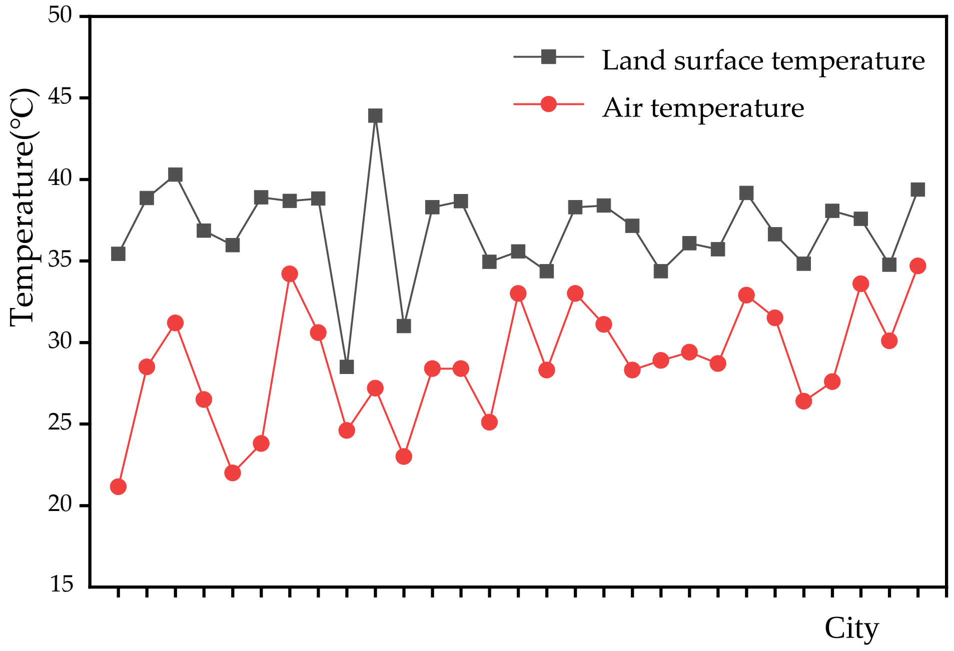

Figure S1. The comparison of the air temperature and the land surface temeparture in 29 cities.

Table S3. Information and cooling effect of 502 urban parks in the study.

Number	Urban park name	Location	Climatic region	PCI/℃	PCA/ha
1	Saihantala Ecological Park	Baotou	arid/semi-arid climate	2.64	118.78
2	Model Worker Park	Baotou	arid/semi-arid climate	3.58	25.24
3	Fuqiang Community Sports Park	Baotou	arid/semi-arid climate	0.83	13.86
4	Rare Earth Park	Baotou	arid/semi-arid climate	1.98	14.50
5	Children Park	Baotou	arid/semi-arid climate	4.17	37.93
6	Baotou amusement park	Baotou	arid/semi-arid climate	3.84	25.32
7	Jingping park	Baotou	arid/semi-arid climate	1.36	29.27
8	Jinxiu Park	Baotou	arid/semi-arid climate	3.72	18.23
9	Citizen Park	Baotou	arid/semi-arid climate	3.72	88.57
10	Baotou Legal Park	Baotou	arid/semi-arid climate	3.84	22.15
11	Qingshan Park	Baotou	arid/semi-arid climate	6.60	31.75
12	Friendship Park	Baotou	arid/semi-arid climate	2.19	11.43
13	North Weapon City Park	Baotou	arid/semi-arid climate	2.95	48.57
14	Yiji Park	Baotou	arid/semi-arid climate	3.49	36.88
15	Yingbin park	Baotou	arid/semi-arid climate	3.68	55.28
16	Jinlin Park	Baotou	arid/semi-arid climate	3.96	46.15
17	Wusutu Park	Baotou	arid/semi-arid climate	3.30	33.83
18	Citizen Park	Beijing	semi-humid climate	1.27	18.85
19	Baliqiao Park	Beijing	semi-humid climate	1.43	25.43
20	Wukesong Park	Beijing	semi-humid climate	1.90	59.45
21	Side Park	Beijing	semi-humid climate	2.13	17.28
22	Shijingshan Sculpture Park	Beijing	semi-humid climate	2.18	15.49
23	Ming Great Wall Heritage Park	Beijing	semi-humid climate	2.31	48.05
24	Dongsheng Bajia Country Park	Beijing	semi-humid climate	2.33	35.48
25	Jiulongkou Leisure Park	Beijing	semi-humid climate	2.51	24.07
26	Dongfeng Park	Beijing	semi-humid climate	2.72	37.73
27	Chaoyang Park	Beijing	semi-humid climate	2.92	67.05
28	Majiawan Wetland Park	Beijing	semi-humid climate	2.93	63.22
29	Jiangfu Park	Beijing	semi-humid climate	2.97	56.70
30	Laojuntang Park	Beijing	semi-humid climate	3.02	51.63
31	Wangxing Lake Park	Beijing	semi-humid climate	3.10	26.61
32	Heishan Park	Beijing	semi-humid climate	3.22	19.43
33	Nanhaizi Park	Beijing	semi-humid climate	3.57	77.52
34	Haitang Park	Beijing	semi-humid climate	3.58	31.60
35	Jingcheng Forest Park	Beijing	semi-humid climate	3.63	38.94
36	Eastern Suburb Wetland Park	Beijing	semi-humid climate	3.75	110.07
37	Hongbo Park	Beijing	semi-humid climate	3.86	46.89
38	Fengyi Park	Beijing	semi-humid climate	3.91	39.29
39	Laoshan City Leisure Park	Beijing	semi-humid climate	3.92	47.63
40	Riverside Century Square Park	Beijing	semi-humid climate	3.94	22.62
41	Wangjing Park	Beijing	semi-humid climate	4.17	39.85

42	Rending Lake Park	Beijing	semi-humid climate	4.28	25.66
43	Youth Lake Park	Beijing	semi-humid climate	4.85	24.41
44	Xihui Park	Beijing	semi-humid climate	4.93	69.83
45	Yuetan Park	Beijing	semi-humid climate	4.94	62.54
46	Niantan Park	Beijing	semi-humid climate	5.34	61.25
47	Liuyin Park	Beijing	semi-humid climate	5.38	24.86
48	Xinglong Country Park	Beijing	semi-humid climate	5.41	34.05
49	Yuyuantan park	Beijing	semi-humid climate	5.61	74.91
50	Yuanmingyuan Heritage Park	Beijing	semi-humid climate	6.12	122.58
51	Zizhuyuan Park	Beijing	semi-humid climate	6.46	49.89
52	Taoranting Park	Beijing	semi-humid climate	6.89	70.29
53	Zhongta Park	Beijing	semi-humid climate	2.55	10.87
54	Linglong Park	Beijing	semi-humid climate	2.97	31.82
55	Shuangxiu Park	Beijing	semi-humid climate	3.69	21.89
56	Tuanjie Lake Park	Beijing	semi-humid climate	4.50	24.72
57	Red scarf park	Beijing	semi-humid climate	5.48	27.15
58	Temple of Heaven Park	Beijing	semi-humid climate	5.62	84.84
59	Wanfangting Community Park	Beijing	semi-humid climate	3.38	20.00
60	Xinfadi Park	Beijing	semi-humid climate	3.52	33.22
61	Huilongguan Park	Beijing	semi-humid climate	2.21	20.95
62	Dongba Country Park	Beijing	semi-humid climate	3.25	107.52
63	Beihai Park	Beijing	semi-humid climate	7.67	37.48
64	Lotus Pond Park	Beijing	semi-humid climate	7.61	30.21
65	Longtan Park	Beijing	semi-humid climate	4.93	38.31
66	Tianyuan Park	Beijing	semi-humid climate	3.34	25.14
67	Boda Park	Beijing	semi-humid climate	4.37	29.05
68	Yushuzhuang Park	Beijing	semi-humid climate	3.87	23.03
69	People's Park	Chengdou	humid climate	4.28	26.79
70	Cultural Park	Chengdou	humid climate	1.97	16.86
71	Xinhua Park	Chengdou	humid climate	3.87	22.46
72	Tazishan Park	Chengdou	humid climate	2.21	37.38
73	Wangjianglou Park	Chengdou	humid climate	3.82	65.17
74	East Lake Park	Chengdou	humid climate	6.25	60.03
75	Shahe Dongli Cuihu Park	Chengdou	humid climate	2.90	50.88
76	Jinjiang Park	Chengdou	humid climate	1.69	21.13
77	Qinglong Lake Wetland Park	Chengdou	humid climate	4.90	122.77
78	Guixi Ecological Park	Chengdou	humid climate	1.18	33.72
79	Jincheng Park	Chengdou	humid climate	1.07	28.29
80	American Fashion Sports Park	Chengdou	humid climate	0.73	30.70
81	Jinsha Heritage Park	Chengdou	humid climate	3.02	25.29
82	Jinsha Riverside Park	Chengdou	humid climate	2.09	29.17
83	Tea Culture Park	Chengdou	humid climate	1.77	25.25
84	Shahe Park	Chengdou	humid climate	3.92	100.93
85	Chengdu Yongling Museum Park	Chengdou	humid climate	3.60	28.13

86	Jiaojinshan Mountain Park	Dalian	semi-humid climate	1.82	37.12
87	Pearl Park	Dalian	semi-humid climate	2.21	26.76
88	Dingshan Park	Dalian	semi-humid climate	2.21	19.44
89	Zhongshan Park	Dalian	semi-humid climate	3.14	30.72
90	Children Park	Dalian	semi-humid climate	3.14	23.33
91	Zhoubei Park	Dalian	semi-humid climate	3.35	15.74
92	Fumin Park	Dalian	semi-humid climate	3.43	15.79
93	Shuxiangyuan Education Park	Dalian	semi-humid climate	3.60	51.07
94	Qianguan Wetland Park	Dalian	semi-humid climate	3.68	50.64
95	Guangming Park	Dalian	semi-humid climate	4.02	13.76
96	Shanri Health Park	Dalian	semi-humid climate	4.09	11.49
97	Zaoyuan Park	Dalian	semi-humid climate	4.33	29.15
98	Beichen Park	Dalian	semi-humid climate	4.47	70.05
99	Fuguo Park	Dalian	semi-humid climate	4.48	65.41
100	Pool Mountain Park	Dalian	semi-humid climate	4.72	50.91
101	Niutou Mountain Park	Dalian	semi-humid climate	5.11	31.05
102	Battery Park	Dalian	semi-humid climate	5.12	60.47
103	Yuhe Ecological Park	Datong	arid/semi-arid climate	4.42	69.54
104	Mingtang Park	Datong	arid/semi-arid climate	2.44	13.83
105	Zhijiabao Forest Park	Datong	arid/semi-arid climate	4.36	46.94
106	Shilihe Forest Park	Datong	arid/semi-arid climate	3.33	46.77
107	Green Axis Park	Datong	arid/semi-arid climate	1.13	47.80
108	Wedding Culture Park	Ordos	arid/semi-arid climate	2.69	43.79
109	Ningxin Community Park	Ordos	arid/semi-arid climate	1.39	17.59
110	Grassland Love Park	Ordos	arid/semi-arid climate	0.24	17.75
111	Kangbashi Central Park	Ordos	arid/semi-arid climate	2.30	39.92
112	Kangxinyuan Community Park	Ordos	arid/semi-arid climate	3.35	24.13
113	Ajinai Park	Ordos	arid/semi-arid climate	4.22	23.89
114	Limin Square Park	Ordos	arid/semi-arid climate	1.53	10.84
115	Tiexi Park	Ordos	arid/semi-arid climate	3.52	30.80
116	Delta Park	Ordos	arid/semi-arid climate	2.41	23.72
117	Holiday Park	Ordos	arid/semi-arid climate	2.22	48.08
118	Diligence Park	Ordos	arid/semi-arid climate	2.16	15.36
119	Mother's Park	Ordos	arid/semi-arid climate	2.28	19.98
120	Shengdeng Park	Ordos	arid/semi-arid climate	1.52	15.61
121	Spa park	Fuzhou	humid climate	2.47	62.10
122	Yushan Scenic Area	Fuzhou	humid climate	2.53	17.26
123	Wushan Scenic Area	Fuzhou	humid climate	2.20	37.88
124	Jinniushan Park	Fuzhou	humid climate	3.21	16.37
125	Aofeng Park	Fuzhou	humid climate	1.39	18.40
126	Chang'an Mountain Park	Fuzhou	humid climate	3.75	35.95
127	Feifengshan Smart Park	Fuzhou	humid climate	4.84	64.25
128	Fuzhou Children's Park	Fuzhou	humid climate	0.88	35.39
129	Luohan Mountain Park	Fuzhou	humid climate	4.09	29.38

130	Changshan Lake Park	Fuzhou	humid climate	3.41	17.49
131	Bingxin Park	Fuzhou	humid climate	1.40	18.90
132	Gaogaishan Park	Fuzhou	humid climate	7.90	172.42
133	Niugang Mountain Park	Fuzhou	humid climate	2.93	67.83
134	Pingshan Park	Fuzhou	humid climate	2.68	45.26
135	West Lake Park	Fuzhou	humid climate	4.84	98.35
136	Northern Suburb Park	Jiuquan	arid/semi-arid climate	1.67	32.75
137	Jiuquan Park	Jiuquan	arid/semi-arid climate	5.20	27.07
138	Space Park	Jiuquan	arid/semi-arid climate	3.27	59.77
139	New World Community Park	Jiuquan	arid/semi-arid climate	2.95	19.33
140	Kangxin Community Park	Jiuquan	arid/semi-arid climate	3.59	20.26
141	Yueliangwan Community Park	Jiuquan	arid/semi-arid climate	3.59	25.10
142	Children's Park	Jiuquan	arid/semi-arid climate	1.30	9.41
143	Shenzhou Pearl Community Park	Jiuquan	arid/semi-arid climate	2.52	16.36
144	Indus Park	Jiuquan	arid/semi-arid climate	3.08	22.98
145	Zijing Community Park	Jiuquan	arid/semi-arid climate	1.97	34.03
146	Zhaoqi Community Park	Jiuquan	arid/semi-arid climate	1.21	16.94
147	East Lake Park	Jiayuguan	arid/semi-arid climate	8.54	70.02
148	Wuyi Community Park	Jiayuguan	arid/semi-arid climate	2.71	17.67
149	Forest Park	Jiayuguan	arid/semi-arid climate	7.07	26.46
150	Xiongguan Park	Jiayuguan	arid/semi-arid climate	4.65	19.89
151	Yingbin Lake Tourist Park	Jiayuguan	arid/semi-arid climate	6.64	55.20
152	Xiaogang Park	Guangzhou	humid climate	5.26	37.84
153	Zhuangtou Park	Guangzhou	humid climate	5.39	21.22
154	The people's Park	Guangzhou	humid climate	4.16	27.22
155	Liwan Lake Park	Guangzhou	humid climate	6.68	38.75
156	Youth Park	Guangzhou	humid climate	2.06	9.50
157	Liuhuahu park	Guangzhou	humid climate	5.63	52.08
158	Yuexiu Park	Guangzhou	humid climate	5.34	65.20
159	Huanghuagang Park	Guangzhou	humid climate	4.44	31.62
160	Dongfeng Park	Guangzhou	humid climate	2.03	13.74
161	Yanling Park	Guangzhou	humid climate	7.26	51.69
162	Peak Park	Guangzhou	humid climate	5.44	19.13
163	Tianhe park	Guangzhou	humid climate	4.62	52.37
164	Pearl River Park	Guangzhou	humid climate	4.12	55.48
165	Zengbu Park	Guangzhou	humid climate	3.21	21.69
166	Pengjiamu Park	Guangzhou	humid climate	4.93	16.71
167	Children's Park	Guangzhou	humid climate	3.16	24.03
168	Dongshan Lake Park	Guangzhou	humid climate	3.46	42.92
169	Xiangjiang Park	Harbin	semi-humid climate	1.34	30.80
170	Eurasian Window Park	Harbin	semi-humid climate	1.40	26.23
171	Yuyang Park	Harbin	semi-humid climate	1.43	23.54
172	Pine and cypress ecological park	Harbin	semi-humid climate	1.46	22.93
173	China Pavilion Garden	Harbin	semi-humid climate	1.58	51.32

174	Lilac Park	Harbin	semi-humid climate	1.59	35.94
175	Runfu Park	Harbin	semi-humid climate	1.88	23.61
176	Sports Park	Harbin	semi-humid climate	2.19	26.46
177	New District Central Park	Harbin	semi-humid climate	2.44	63.48
178	Qingbin Park	Harbin	semi-humid climate	2.81	21.04
179	Harbin Cultural Park	Harbin	semi-humid climate	2.84	32.92
180	Jingyu Park	Harbin	semi-humid climate	2.84	18.82
181	Forest Botanical Garden	Harbin	semi-humid climate	2.86	203.12
182	Labor Park	Harbin	semi-humid climate	2.95	57.51
183	Xing'an Road Park	Harbin	semi-humid climate	2.96	46.23
184	Ancient pear orchard	Harbin	semi-humid climate	3.08	25.03
185	Shangzhi Park	Harbin	semi-humid climate	3.10	15.78
186	Jianguo Park	Harbin	semi-humid climate	3.20	14.65
187	zhaolin park	Harbin	semi-humid climate	3.30	23.93
188	Children's Park	Harbin	semi-humid climate	3.66	53.05
189	Guanghan Park	Harbin	semi-humid climate	4.01	36.11
190	Jiangyi Park	Hangzhou	humid climate	3.10	13.02
191	Xin'an Park	Hangzhou	humid climate	2.54	17.43
192	Yuji Park	Hangzhou	humid climate	3.26	14.77
193	Jiangsi Park	Hangzhou	humid climate	3.58	21.02
194	Chengdong Park	Hangzhou	humid climate	4.82	71.59
195	Hangzhou Chengbei Sports Park	Hangzhou	humid climate	3.71	53.95
196	Jialuyuan Park	Hangzhou	humid climate	3.33	23.86
197	Ducheng Ecological Park	Hangzhou	humid climate	4.29	47.23
198	Caozhuang Park	Hangzhou	humid climate	3.18	16.50
199	Beishan Park	Hangzhou	humid climate	6.57	70.26
200	Nanhu Wetland Park	Huhhot	arid/semi-arid climate	1.33	194.92
201	Xilin Park	Hohhot	arid/semi-arid climate	2.87	30.89
202	Shiqi Park	Hohhot	arid/semi-arid climate	3.78	29.85
203	Wulafu Park	Hohhot	arid/semi-arid climate	4.78	40.56
204	Xinhua Park	Hohhot	arid/semi-arid climate	3.60	21.82
205	Arboretum	Hohhot	arid/semi-arid climate	5.20	33.38
206	Chilechuan Park	Hohhot	arid/semi-arid climate	2.18	19.77
207	Princess House Park	Hohhot	arid/semi-arid climate	2.98	19.17
208	Yuan Culture Park	Hohhot	arid/semi-arid climate	0.42	9.84
209	North Suburban Park	Hohhot	arid/semi-arid climate	3.61	20.35
210	Riverside park	Hohhot	arid/semi-arid climate	3.60	17.24
211	Suyala Park	Hohhot	arid/semi-arid climate	2.72	12.59
212	Genghis Khan Park	Hohhot	arid/semi-arid climate	3.12	31.60
213	Gaoleqi Park	Hohhot	arid/semi-arid climate	0.75	11.34
214	Beishan Park	Hohhot	arid/semi-arid climate	1.56	16.24
215	Yantan Park	Lanzhou	arid/semi-arid climate	3.15	36.37
216	Lanzhou Botanical Garden	Lanzhou	arid/semi-arid climate	5.26	48.34
217	Renshou Mountain Park	Lanzhou	arid/semi-arid climate	2.18	54.92

218	Donggang Small Amusement Park	Lanzhou	arid/semi-arid climate	0.85	8.51
219	Xiaoxihu Park	Lanzhou	arid/semi-arid climate	1.84	13.14
220	Qilihe Community Park	Lanzhou	arid/semi-arid climate	4.18	16.19
221	Jianning Road Community Park	Lanzhou	arid/semi-arid climate	2.72	20.77
222	Nanchang Zoo	Nanchang	humid climate	2.44	85.18
223	Bayi Park	Nanchang	humid climate	4.28	35.62
224	People's Park	Nanchang	humid climate	3.34	32.53
225	Ruziting Park	Nanchang	humid climate	2.16	21.15
226	Jiulonghu Wetland Park	Nanchang	humid climate	5.88	73.47
227	Jinhu Park	Nanchang	humid climate	4.56	44.64
228	Chengbi Lake Park	Nanchang	humid climate	4.75	51.61
229	Tianxiang Garden	Nanchang	humid climate	2.91	38.35
230	Huanyu Vientiane Park	Nanchang	humid climate	1.91	42.10
231	Libu Lake Park	Nanchang	humid climate	3.60	40.79
232	Malanwei Wetland Park	Nanchang	humid climate	2.48	25.62
233	Universal Park	Nanchang	humid climate	3.29	42.88
234	Stone Town Park	Nanjing	humid climate	2.21	25.37
235	Qingliangshan Park	Nanjing	humid climate	4.04	27.11
236	Wulongtan Park	Nanjing	humid climate	2.71	18.72
237	Mochou Lake Park	Nanjing	humid climate	6.94	38.22
238	Guling Park	Nanjing	humid climate	4.38	37.80
239	River Street Amusement Park	Nanjing	humid climate	2.20	21.37
240	Qiqiaoweng Wetland Park	Nanjing	humid climate	3.22	80.54
241	Jiangning Xintiandi Park	Nanjing	humid climate	1.26	14.63
242	Yuhuatai Park	Nanjing	humid climate	4.38	88.76
243	Hexi City Ecological Park	Nanjing	humid climate	2.05	15.03
244	Lotus Lake Park	Nanjing	humid climate	4.68	40.18
245	Stone Carved Lake Park	Nanjing	humid climate	5.13	39.06
246	Hongshan Forest Zoo	Nanjing	humid climate	5.22	64.46
247	Taipingshan Park	Nanjing	humid climate	5.22	58.53
248	Huadian Xiyuan Mountain Park	Nanjing	humid climate	3.64	36.15
249	Pukou Park	Nanjing	humid climate	4.85	33.47
250	Bailuzhou Park	Nanjing	humid climate	3.76	26.06
251	Baota Mountain Forest Park	Nanjing	humid climate	3.76	35.94
252	Xiaoniutou Mountain Park	Nanjing	humid climate	3.52	34.85
253	Gaoqiao Park	Shanghai	humid climate	2.72	18.24
254	Gangcheng Park	Shanghai	humid climate	2.27	19.83
255	Minxing Park	Shanghai	humid climate	2.24	23.60
256	Huangxing Park	Shanghai	humid climate	4.16	50.02
257	Yangpu Park	Shanghai	humid climate	6.22	35.85
258	Jiangpu Park	Shanghai	humid climate	2.44	9.42
259	Peace Park	Shanghai	humid climate	3.89	40.18
260	Luxun Park	Shanghai	humid climate	4.33	37.36
261	Zhabei Park	Shanghai	humid climate	3.35	35.44

262	Daning Tulip Park	Shanghai	humid climate	3.17	51.72
263	Century Park	Shanghai	humid climate	2.41	54.32
264	Pudong Jinhai Wetland Park	Shanghai	humid climate	3.01	118.18
265	Kangqiao Ecological Park	Shanghai	humid climate	2.19	47.01
266	Great North China Park	Shanghai	humid climate	1.66	19.60
267	Caoxi Park	Shanghai	humid climate	2.05	18.02
268	Dream Park	Shanghai	humid climate	3.93	52.41
269	Xujiahui Park	Shanghai	humid climate	3.34	44.63
270	Shanghai Botanical Garden	Shanghai	humid climate	4.06	87.85
271	Li'an Park	Shanghai	humid climate	3.32	65.87
272	Minhang Sports Park	Shanghai	humid climate	2.39	65.43
273	Guangfulin Country Park	Shanghai	humid climate	2.48	58.68
274	Kunxiu Lake Wetland Park	Shanghai	humid climate	1.10	45.53
275	Sixian Park	Shanghai	humid climate	3.14	44.15
276	Jing'an Sculpture Park	Shanghai	humid climate	1.93	16.17
277	Changshou Park	Shanghai	humid climate	4.00	14.50
278	Changfeng Park	Shanghai	humid climate	3.97	56.53
279	Zhongshan Park	Shanghai	humid climate	3.03	38.33
280	Tianshan Park	Shanghai	humid climate	3.78	42.24
281	New Hongqiao Central Garden	Shanghai	humid climate	3.21	25.45
282	Jiamei Greenland Park	Shanghai	humid climate	2.08	53.76
283	Gucun Park	Shanghai	humid climate	2.81	130.00
284	Shawan Park	Shenzhen	humid climate	3.26	19.81
285	Tiezaishan Park	Shenzhen	humid climate	7.19	101.60
286	Liutang Park	Shenzhen	humid climate	4.67	13.30
287	Bao'an Park	Shenzhen	humid climate	4.30	48.08
288	Lingzhi Park	Shenzhen	humid climate	1.81	13.27
289	Xin'an Park	Shenzhen	humid climate	1.37	12.03
290	Zhongshan Park	Shenzhen	humid climate	3.58	41.15
291	Lixiang Park	Shenzhen	humid climate	3.21	64.49
292	Dasha River Park	Shenzhen	humid climate	5.07	38.92
293	Shigushan Park	Shenzhen	humid climate	4.83	31.61
294	Yanhan Mountain Country Park	Shenzhen	humid climate	4.47	80.18
295	OCT National Wetland Park	Shenzhen	humid climate	4.96	55.05
296	Lotus Hill Park	Shenzhen	humid climate	5.46	69.17
297	bijiashan park	Shenzhen	humid climate	5.68	68.12
298	Shenzhen Central Park	Shenzhen	humid climate	2.33	95.14
299	Lizhi Park	Shenzhen	humid climate	5.02	55.04
300	People's Park	Shenzhen	humid climate	4.20	35.01
301	Children's Park	Shenzhen	humid climate	1.82	19.81
302	Cuizhu Park	Shenzhen	humid climate	6.96	45.42
303	Honghu Park	Shenzhen	humid climate	6.57	98.84
304	Wailing Park	Shenzhen	humid climate	6.90	88.97
305	Honggang Park	Shenzhen	humid climate	3.33	22.20

306	Huanggang Park	Shenzhen	humid climate	6.35	54.89
307	Songgang Park	Shenzhen	humid climate	4.61	129.92
308	Honghuashan Park	Shenzhen	humid climate	5.65	39.96
309	Longhua Park	Shenzhen	humid climate	5.41	21.06
310	Shiyaling Park	Shenzhen	humid climate	6.32	50.29
311	Vanke Community Park	Shenyang	semi-humid climate	0.22	3.11
312	Bainiao Park	Shenyang	semi-humid climate	3.27	22.99
313	Construction Park	Shenyang	semi-humid climate	3.32	25.27
314	Riverside Park	Shenyang	semi-humid climate	3.64	27.06
315	Nujiang Park	Shenyang	semi-humid climate	3.72	65.36
316	Labor Community Park	Shenyang	semi-humid climate	4.34	24.70
317	Beita Park	Shenyang	semi-humid climate	4.51	46.58
318	Youth Park	Shenyang	semi-humid climate	4.63	38.09
319	Tiexi Forest Park	Shenyang	semi-humid climate	4.71	57.32
320	Labor Park	Shenyang	semi-humid climate	4.88	57.74
321	South Lake Park	Shenyang	semi-humid climate	5.24	58.57
322	Bayi Park	Shenyang	semi-humid climate	5.37	37.52
323	Huanghai Park	Shenyang	semi-humid climate	5.40	51.16
324	Wanliutang Park	Shenyang	semi-humid climate	5.50	69.87
325	Zhongshan Park	Shenyang	semi-humid climate	5.55	26.17
326	Wanquan Park	Shenyang	semi-humid climate	5.68	60.08
327	Twin Towers Park	Taiyuan	semi-humid climate	1.16	13.38
328	Riverside Park	Taiyuan	semi-humid climate	1.24	7.53
329	Jinqiao Park	Taiyuan	semi-humid climate	1.25	11.60
330	Taiyuan Botanical Garden	Taiyuan	semi-humid climate	1.98	61.97
331	Peace Park	Taiyuan	semi-humid climate	2.49	34.93
332	Yifen Park	Taiyuan	semi-humid climate	2.60	24.71
333	Nanhaizi Park	Taiyuan	semi-humid climate	2.74	15.21
334	Jinyang Street Park	Taiyuan	semi-humid climate	2.75	33.89
335	Yumenhe Park	Taiyuan	semi-humid climate	2.96	34.88
336	Yinmahe Park	Taiyuan	semi-humid climate	3.03	15.56
337	Jinci Park	Taiyuan	semi-humid climate	4.06	57.12
338	Wenying Park	Taiyuan	semi-humid climate	4.42	22.09
339	Xuefu Park	Taiyuan	semi-humid climate	4.65	28.68
340	Longtan Park	Taiyuan	semi-humid climate	5.22	58.20
341	Yingze Park	Taiyuan	semi-humid climate	5.83	66.14
342	Nanpu park	Tianjin	semi-humid climate	0.86	7.39
343	Leaf Park	Tianjin	semi-humid climate	1.14	6.93
344	Fumin Park	Tianjin	semi-humid climate	1.18	18.73
345	Zhongshan Park	Tianjin	semi-humid climate	2.18	7.49
346	Nankai Park	Tianjin	semi-humid climate	2.22	25.43
347	Huaxia Future Children's Park	Tianjin	semi-humid climate	2.31	10.21
348	Xiangyu Park	Tianjin	semi-humid climate	2.82	25.16
349	Qiaoyuan Park	Tianjin	semi-humid climate	3.34	32.62

350	Xigu Park	Tianjin	semi-humid climate	3.39	42.47
351	Binyue Park	Tianjin	semi-humid climate	3.49	34.34
352	Nancuiping Park	Tianjin	semi-humid climate	3.60	25.92
353	People's Park	Tianjin	semi-humid climate	3.62	20.34
354	Hedong Park	Tianjin	semi-humid climate	3.68	29.01
355	Xinli Country Park	Tianjin	semi-humid climate	3.75	75.35
356	Beining Park	Tianjin	semi-humid climate	4.10	40.67
357	Ergong Park	Tianjin	semi-humid climate	4.15	33.92
358	Changhong Ecological Park	Tianjin	semi-humid climate	4.57	28.63
359	Meijiang Park	Tianjin	semi-humid climate	4.68	78.47
360	Children park	Urumqi	arid/semi-arid climate	3.86	27.80
361	People's Park	Urumqi	arid/semi-arid climate	4.40	35.13
362	Jianquan Street Amusement Park	Urumqi	arid/semi-arid climate	3.15	15.10
363	Tuanjie Garden	Urumqi	arid/semi-arid climate	2.66	12.48
364	Urumqi Sports Park	Urumqi	arid/semi-arid climate	2.79	19.19
365	Yan'an Park	Urumqi	arid/semi-arid climate	4.58	36.40
366	South Park	Urumqi	arid/semi-arid climate	4.57	35.90
367	Heping Canal Dazhai Gate Park	Urumqi	arid/semi-arid climate	3.48	40.59
368	Qianshuiwan Ecological Park	Urumqi	arid/semi-arid climate	3.75	26.31
369	Tianshan Park	Urumqi	arid/semi-arid climate	4.09	42.92
370	Ziyang Park	Wuhan	humid climate	5.90	25.40
371	Yellow Crane Tower Park	Wuhan	humid climate	5.23	81.86
372	Shahu Park	Wuhan	humid climate	7.15	107.24
373	Simeitang Park	Wuhan	humid climate	4.66	38.76
374	Wuhan Garden Science Park	Wuhan	humid climate	2.39	22.34
375	Qingshan Park	Wuhan	humid climate	4.00	58.46
376	Daijiahu Park	Wuhan	humid climate	2.32	57.84
377	Yangchun Lake Park	Wuhan	humid climate	5.21	53.78
378	Hongshan Park	Wuhan	humid climate	5.46	61.31
379	Lotus Lake Park	Wuhan	humid climate	4.51	14.47
380	Hanyang Park	Wuhan	humid climate	2.58	11.63
381	Zhongshan Park	Wuhan	humid climate	4.44	40.16
382	Xiaonanhu Park	Wuhan	humid climate	2.23	13.65
383	Baodao Park	Wuhan	humid climate	6.31	39.08
384	Jiefang Park	Wuhan	humid climate	4.85	63.06
385	Lingjiao Lake Park	Wuhan	humid climate	5.65	25.63
386	Houxianghe Park	Wuhan	humid climate	4.02	26.00
387	Wangjiadun Park	Wuhan	humid climate	2.82	22.06
388	Dijiao Park	Wuhan	humid climate	5.14	30.99
389	Evergreen Park	Wuhan	humid climate	3.62	30.60
390	Peace Park	Wuhan	humid climate	2.67	31.53
391	South Trunk Canal Garden	Wuhan	humid climate	3.90	42.91
392	Xingfu Bay Park	Wuhan	humid climate	5.32	27.53
393	Jitou Mountain Park	Wuhan	humid climate	1.83	12.44

394	Sports park	Xi'an	semi-humid climate	1.08	25.57
395	Yunshui Park	Xi'an	semi-humid climate	1.19	27.26
396	Wooden Pagoda Temple Park	Xi'an	semi-humid climate	1.60	52.75
397	Wenjingshan Park	Xi'an	semi-humid climate	2.29	56.60
398	Wenjing Park	Xi'an	semi-humid climate	2.32	20.75
399	Space City Lake Park	Xi'an	semi-humid climate	2.89	22.34
400	Taohuatan Park	Xi'an	semi-humid climate	2.98	56.64
401	Tang Paradise	Xi'an	semi-humid climate	3.02	38.33
402	Changle Park	Xi'an	semi-humid climate	3.02	37.96
403	Fengqing Park	Xi'an	semi-humid climate	3.42	29.70
404	Qujiangchi Heritage Park	Xi'an	semi-humid climate	3.61	50.23
405	Martyrs' Cemetery	Xi'an	semi-humid climate	3.69	32.57
406	Weiyang Lake Park	Xi'an	semi-humid climate	3.81	28.92
407	Xingqing Palace Park	Xi'an	semi-humid climate	3.91	40.56
408	Lianhu Park	Xi'an	semi-humid climate	4.08	26.62
409	Revolution Park	Xi'an	semi-humid climate	4.30	43.17
410	Forest Park	Yinchuan	arid/semi-arid climate	3.92	69.13
411	Yuxiu Park	Yinchuan	arid/semi-arid climate	2.76	69.47
412	Mingcui Lake Wetland Park	Yinchuan	arid/semi-arid climate	2.68	104.81
413	Tanglai Park	Yinchuan	arid/semi-arid climate	4.34	31.48
414	Yange Lake Park	Yinchuan	arid/semi-arid climate	4.76	19.53
415	Lijing Lake Park	Yinchuan	arid/semi-arid climate	5.15	23.51
416	Huayan Lake Park	Yinchuan	arid/semi-arid climate	4.82	48.25
417	Liberation Park	Yinchuan	arid/semi-arid climate	5.83	52.85
418	Spring Fulled Garden	Yinchuan	arid/semi-arid climate	2.64	14.38
419	Shuangqukou Park	Yinchuan	arid/semi-arid climate	3.25	36.38
420	Lailong Park	Yinchuan	arid/semi-arid climate	2.94	47.83
421	Lizi Park	Yinchuan	arid/semi-arid climate	2.46	35.49
422	Xingqing Park	Yinchuan	arid/semi-arid climate	3.95	17.25
423	Wenchang Park	Yinchuan	arid/semi-arid climate	3.84	36.03
424	Ningcui Park	Yinchuan	arid/semi-arid climate	2.46	33.30
425	Bayi Sports Park	Yinchuan	arid/semi-arid climate	4.70	19.12
426	Bibo Park	Yinchuan	arid/semi-arid climate	3.78	51.36
427	Country Park	Yinchuan	arid/semi-arid climate	1.66	15.08
428	Zidongyuan Park	Yinchuan	arid/semi-arid climate	1.44	6.15
429	Lanradial Lake Park	Changchun	semi-humid climate	1.11	16.71
430	Beihai Park	Changchun	semi-humid climate	1.57	46.45
431	Junzilan Park/Clivia Park	Changchun	semi-humid climate	2.52	17.00
432	Furong Community Park	Changchun	semi-humid climate	3.18	26.55
433	Tianxi Park	Changchun	semi-humid climate	3.42	46.35
434	Jinjiang Park	Changchun	semi-humid climate	3.64	32.81
435	Central Park	Changchun	semi-humid climate	3.70	26.95
436	Daishan Park	Changchun	semi-humid climate	4.10	27.33
437	Cultural Ecological Park	Changchun	semi-humid climate	4.26	26.49

438	Friendship Park	Changchun	semi-humid climate	4.34	34.92
439	Victory Park	Changchun	semi-humid climate	5.28	29.05
440	Labor Park	Changchun	semi-humid climate	5.35	21.46
441	Changchun Deyuan Park	Changchun	semi-humid climate	5.67	29.38
442	Xiufengshan Park	Changcha	humid climate	2.03	50.01
443	Vanke City Community Park	Changcha	humid climate	0.48	10.40
444	South Park	Changcha	humid climate	1.95	30.69
445	Changsha Shawan Park	Changcha	humid climate	1.29	76.20
446	Heili Community Park	Changcha	humid climate	0.53	6.62
447	Xiangu Mountain Park	Changcha	humid climate	3.37	115.03
448	Lion Mountain Park	Changcha	humid climate	1.52	22.83
449	Yanghu National Wetland Park	Changcha	humid climate	1.32	110.56
450	Wangyue Park	Changcha	humid climate	1.71	38.51
451	West Lake Park	Changcha	humid climate	2.71	62.54
452	Bafang Park	Changcha	humid climate	3.22	33.25
453	Mountain Park	Changcha	humid climate	2.57	17.51
454	Vientiane Park	Changcha	humid climate	0.90	21.17
455	Lugu Park	Changcha	humid climate	2.60	37.20
456	Meiling Park	Changcha	humid climate	2.87	49.09
457	Haitang Park	Changcha	humid climate	2.34	18.97
458	Xiangfu Cultural Park	Changcha	humid climate	1.33	19.56
459	Yuehu Park	Changcha	humid climate	2.64	33.73
460	Xingsha Cultural Park	Changcha	humid climate	5.29	46.47
461	Shuangyong Park	Changcha	humid climate	3.24	52.70
462	Songya Lake Wetland Park	Changcha	humid climate	3.49	95.50
463	Xianglu Villa Park	Changcha	humid climate	3.61	43.54
464	Green City Park	Zhengzhou	semi-humid climate	0.98	11.76
465	Shuangxiu Park	Zhengzhou	semi-humid climate	1.15	13.13
466	Shanggangyang Ruins Park	Zhengzhou	semi-humid climate	1.23	30.12
467	Cultural Park	Zhengzhou	semi-humid climate	1.56	22.22
468	Zheng Xin Park	Zhengzhou	semi-humid climate	1.65	28.15
469	Wenbo Park	Zhengzhou	semi-humid climate	1.76	22.05
470	Lvyin Park	Zhengzhou	semi-humid climate	1.93	15.39
471	South Ring Park	Zhengzhou	semi-humid climate	2.16	26.68
472	Shangdu Heritage Park	Zhengzhou	semi-humid climate	2.32	35.49
473	Jingxiu Park	Zhengzhou	semi-humid climate	2.64	48.93
474	Wuyi Park	Zhengzhou	semi-humid climate	2.83	18.12
475	Bishagang Park	Zhengzhou	semi-humid climate	2.90	42.03
476	Riverside Park	Zhengzhou	semi-humid climate	3.02	28.74
477	Zijing Mountain Park	Zhengzhou	semi-humid climate	3.13	39.81
478	Longzi Lake Park	Zhengzhou	semi-humid climate	3.61	127.05
479	Rose Park	Zhengzhou	semi-humid climate	3.64	37.27
480	Zhengzhou Zoo	Zhengzhou	humid climate	3.98	24.61
481	People's Park	Zhengzhou	humid climate	4.61	62.52

482	Pipa Mountain Park	Chongqing	humid climate	1.93	17.33
483	Rongqiao Park	Chongqing	humid climate	1.63	29.64
484	Chongqing Zoo	Chongqing	humid climate	3.91	64.47
485	Caiyun Lake Wetland Park	Chongqing	humid climate	4.07	154.78
486	Dadukou Park	Chongqing	humid climate	3.45	23.61
487	Huayan Scenic Area	Chongqing	humid climate	5.45	78.48
488	Banshan Park	Chongqing	humid climate	2.65	35.44
489	Shimen Park	Chongqing	humid climate	2.34	26.12
490	Hong'en Temple Forest Park	Chongqing	humid climate	2.61	58.78
491	Chongqing Flower Garden	Chongqing	humid climate	2.35	30.03
492	Dalongshan Park	Chongqing	humid climate	1.83	15.59
493	Panxi River Park	Chongqing	humid climate	2.84	26.61
494	Bailin Park	Chongqing	humid climate	1.95	41.61
495	Yubei Longtou Temple Park	Chongqing	humid climate	2.28	70.67
496	Jinshan park	Chongqing	humid climate	3.41	77.54
497	Palm Springs Ecological Park	Chongqing	humid climate	2.36	29.11
498	Baosheng Lake Park	Chongqing	humid climate	6.42	114.42
499	Muyu-Stone Park	Chongqing	humid climate	5.46	36.83
500	Guanyin Park	Chongqing	humid climate	3.55	64.70
501	Bi-chun Park	Chongqing	humid climate	4.53	60.69
502	Taoyuan Park	Chongqing	humid climate	4.41	57.24

Table S4. The Pearson correlation coefficients between park cooling effect and impact factors in three climates climatic regions.

**Impact factors**	**arid/semi-arid climates**		**semi-humid climates**		**humid climates**	
	**PCI**	**PCA**	**PCI**	**PCA**	**PCI**	**PCA**
PA	0.0358	0.8054**	0.2109**	0.6862**	0.1908**	0.5958**
LSI	-0.0806	0.4179**	-0.0931	0.4538**	0.1629*	0.5540**
WR	0.3803**	0.3382**	0.4090**	0.2225**	0.2679**	0.0597
WAI	0.3158**	0.3806**	0.3131**	0.2516**	0.2026**	0.2870**
WED	0.3280**	0.2542*	0.3611**	0.1093	0.1813**	-0.0501
BIR	0.3403**	-0.3251**	0.1338	-0.3155**	0.1007	-0.2428**
BGR	-0.3520**	0.3098**	-0.1534*	0.3212**	-0.0823	0.2576**
BWR	-0.0687	0.3119**	-0.0628	0.0579	-0.0806	-0.0098
BGT	0.2731**	-0.1973	0.4272**	-0.0872	0.2895**	-0.0527
DEM	-0.0045	-0.1340	-0.1400	-0.0830	-0.1086	0.0703

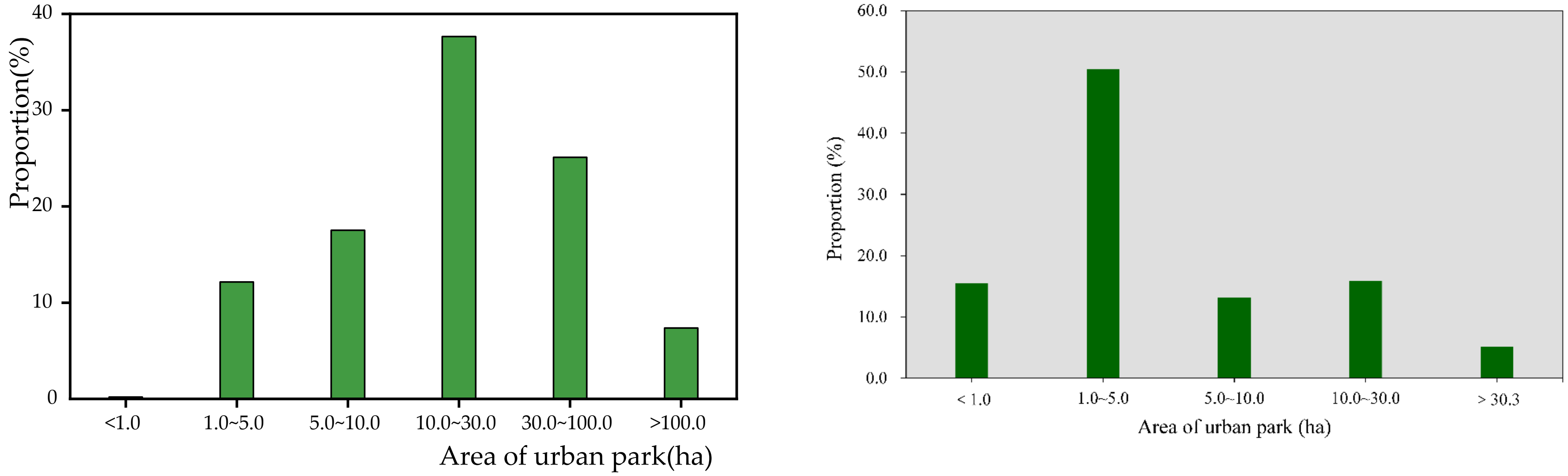

Figure S2. The comparison of area distribution of urban park. The left figure shows the area distribution of urban park in this study, the right figure shows the area of urban park distribution by Geng et al. (2022).

Table S5. The dominance analysis of influencing factors of park cooling effect without waterbodies in humid regions.

Impact factors	Contribution rate [ranking]
PA	59.25% [1]
LSI	5.48% [4]
WR	1.17% [8]
WAI	3.44% [5]
WED	0.45% [10]
BIR	0.95% [9]
BGR	1.21% [7]
BWR	1.32% [6]
BGT	19.47% [2]
DEM	7.27% [3]
